# Evaluation of choroidal thickness measurements in patients with marked nasal septal deviation^☆^

**DOI:** 10.1016/j.bjorl.2018.11.009

**Published:** 2018-12-31

**Authors:** Selin Üstün Bezgin, Taliye Çakabay, Sadık Etka Bayramoğlu, Nihat Sayın, Murat Koçyiğit

**Affiliations:** a Istanbul Kanuni Sultan Süleyman Training and Research Hospital, Department of Otorhinolaryngology, Istanbul, Turkey; b Istanbul Kanuni Sultan Süleyman Training and Research Hospital, Department of Ophthalmology, Istanbul, Turkey

**Keywords:** Nasal septal deviation, Choroidal thickness, Sleep-disordered breathing, Desvio do septo nasal, Espessura da coroide, Distúrbios respiratórios do sono

## Abstract

**Introduction:**

Chronic upper airway obstruction due to marked nasal septal deviation may cause chronic hypoxia. It may change the balance of the sympathetic-parasympathetic system and may affect blood flow in the choroid.

**Objective:**

To assess choroidal thickness measurements of patients with marked nasal septal deviation.

**Methods:**

The patients who had nasal obstruction symptoms diagnosed with marked nasal septal deviation by anterior rhinoscopy and nasal endoscopy and scheduled for septoplasty were included in the study. The control group consisted of age, sex and body mass index-matched healthy individuals. The choroidal measurements at the central fovea and 1000 μm away from the fovea in the nasal and temporal regions were performed using enhanced depth imaging optical coherence tomography.

**Results:**

In the study group, 52 eyes of 26 patients with a mean age of 26.34 ± 8.14 years were examined. In the control group, 52 eyes of 28 healthy individuals with a mean age of 26.69 ± 7.84 years were examined. There was no statistically significant difference in terms of choroidal thickness measurements between the groups (*p* > 0.05).

**Conclusion:**

Our results suggest that marked nasal septal deviation may not lead to significant hypoxia and sympathetic activation, resulting in deterioration of the choroidal blood flow and consequent choroidal thickening.

## Resumo

**Introdução:**

A obstrução crônica das vias aéreas superiores devido a acentuado desvio do septo nasal pode causar hipóxia crônica. Pode alterar o equilíbrio do sistema simpático-parassimpático e afetar o fluxo sanguíneo na coroide.

**Objetivo:**

Avaliar as medidas da espessura da coroide em pacientes com acentuado desvio de septo nasal.

**Método:**

Foram incluídos no estudo pacientes que apresentavam sintomas de obstrução nasal, com diagnóstico de acentuado desvio de septo realizado por rinoscopia anterior e endoscopia nasal, e com septoplastia programada. O grupo controle consistiu de indivíduos saudáveis pareados por idade, sexo e índice de massa corporal. As medidas da coroide na fóvea central e a 1.000 μm da fóvea nas regiões nasal e temporal foram feitas com tomografia de coerência óptica com imagem de profundidade melhorada.

**Resultados:**

No grupo de pacientes, 52 olhos de 26 pacientes com média de 26,34 ± 8,14 anos foram examinados. No grupo controle, 52 olhos de 28 indivíduos saudáveis com média de 26,69 ± 7,84 anos foram examinados. Não houve diferença estatisticamente significante em termos de medidas da espessura da coroide entre os grupos (*p* > 0,05).

**Conclusão:**

Nossos resultados sugerem que desvios do septo nasal acentuados podem não levar à hipóxia significativa e ativação simpática, resultar na deterioração do fluxo sanguíneo coroidal e consequente espessamento da coroide.

## Introduction

Nasal Septal Deviation (NSD) is one of the major causes of upper airway obstruction.^1^ It has be suggested that chronic upper airway obstruction may cause chronic hypoxia, or hypercarbia because of alveolar hypoventilation.^1^, ^2^, ^3^, ^4^ Marked Nasal Septal Deviation (MNSD) may lead to upper airway obstruction chronically and severely.

The choroid, a vascularized structure, is supplied by the posterior ciliary arteries and accounts for approximately 90% of the total ocular blood flow. The choroid's vasculature is the major supply of oxygen and nutrients for the outer retina. The choroidal blood flow may also cool and warm the retina. The choroid, being a vascular structure, can be affected by systemic circumstances such as diabetes, chronic heart failure, and hypertension. Previous studies have reported that increased or decreased choroidal circulation may be associated with abnormal Choroidal Thickness (CT).^5^ The thickest part of normal choroid is under the fovea while thinning exists in the surrounding areas of fovea.^6^ Currently, Enhanced Depth Imaging Optical Coherence Tomography (EDI-OCT) is a relatively new technique and is commonly used as an imaging modality for qualitative and quantitative assessment of the choroid in daily practice.^6^

Many studies in the literature have investigated the effect of Obstructive Sleep Apnea Syndrome (OSAS) on CT.^7^, ^8^, ^9^, ^10^, ^11^ In a meta-analysis evaluating these studies, a decline in choroid thickness was found in the CT of patients with Obstructive Sleep Apnea (OSA).^12^ It was argued that the enhancing effect of recurrent upper respiratory tract obstructions during sleep on sympathetic activity, hypoxia and hypercapnia resulted from deterioration of vascular circulation and choroidal blood flow.

Chronic hypoxia and hypercapnia due to NSD may affect the choroidal blood flow and may change the choroidal thickness. Although several studies have investigated CT in patients with OSAS,^7^, ^8^, ^9^, ^10^, ^11^ to best of our knowledge there is no study investigating CT in patients with NSD. Therefore, in the present study, we aimed to evaluate the CT using EDI-OCT in MNSD patients.

## Methods

### Study population and design

This prospective, case-control study was conducted in the Department of Otolaryngology and Ophthalmology between September 2016 and March 2017. The study was approved by the Clinical Research Ethics Committee (Approval No. 2016.3.9) and conducted in accordance with the principles of the Declaration of Helsinki. A verbal and written informed consent was obtained from each participant.

A detailed otorhinolaryngologic examination including oropharynx, nasal passages, nasopharynx, larynx and hypopharynx regions by flexible fiberoptic endoscope were made. This study included a total of 52 eyes of 26 patients who exhibited nasal obstruction symptoms diagnosed with MNSD by anterior rhinoscopy and nasal endoscopy and scheduled for septoplasty between the ages of 18 and 55 years in our hospital. The control group consisted of 52 eyes of 28 age, sex and body mass index-matched healthy individuals.

### Exclusion criteria

Patients with other Upper Airway Obstruction (UAO) – related conditions (rhinitis, enlarged turbinates, nasal polyposis, adenotonsillary hypertrophy, obstructive sleep apnea) were excluded in this study. In addition, patients with chronic inflammatory disease, autoimmune disease, systemic disease (diabetes mellitus, hypertension, coronary artery disease, cardiac and pulmonary diseases), smoking, and long-term drug use were also excluded from the study. Exclusion criteria were also as follows: high myopia or hyperopia (>+3 or −3 diopters of spherical equivalent), a history of intraocular surgery, ocular trauma, uveitis, any topical medication and systemic disease, and poor image due to unstable fixation.

### Examination protocol and study measurements

All participants underwent a detailed ophthalmic examination including slit-lamp biomicroscopy, tonometry, and fundus examination. All participants also underwent Central Corneal Thickness (CCT) and Axial Length (AL) measurements using an ultrasonic scan. All examinations were performed between 9 A.M. and 12 A.M. All participants were asked not to eat or drink any food containing caffeine for 12 h before examination.

### EDI-OCT measurement

All participants were examined by the Cirrus HD-OCT 4000 (Carl Zeiss Meditec, Inc., Dublin, CA). The scan pattern used on the Zeiss Cirrus, HD 5 line raster, is a 6 mm line consisting of 4096 A-scans. These images were taken with the vitreoretinal interface adjacent to the zero-delay, and were not inverted to bring the choroid adjacent to zero-delay, as image inversion using the Cirrus software results in a low-quality image. The HD 5-line raster has 20 B-scans averaged together without tracking. The procedure for EDI-OCT measurements has been previously described.^6^ The CT was measured from the outer portion of the hyperreflective line corresponding to the Retinal Pigment Epithelium (RPE) of the inner surface of the sclera. The horizontal and vertical sections running through the center of the fovea were selected for further analysis. Thus, the CT measurement was taken at the fovea and 1000 μm away from the fovea in the Nasal (N1) and Temporal (T1) regions. Perpendicular lines were drawn from the posterior edge of the RPE to the choroid/sclera junction using Cirrus HD-OCT software. The images were taken and assessed by two experienced technicians (NS and SEB). Both of them were masked in terms of groups. The macular retinal thickness (central 1000 μm thickness of fovea) was also measured using the Cirrus HD-OCT 4000 (Carl Zeiss Meditec, Inc., Dublin, CA).

### Statistical analysis

Statistical analysis was performed using the Statistical Package for the Social Sciences (SPSS) version 18 software (SPSS Inc., Chicago, IL, USA). Descriptive data were expressed in mean ± standard deviation. The normality of the data was confirmed by the Kolmogorov-Smirnov *Z* test. An independent *t*-test and Mann Whitney-*U* were used to compare variables among groups. A *p*-value of <0.05 was considered statistically significant.

## Results

In the group with MNSD, 52 eyes of 26 patients (male-to-female ratio: 44/8) with a mean age of 26.34 ± 8.14 years were evaluated. In the control group, 52 eyes of 28 healthy individuals (male-to-female ratio: 41/11) with a mean age of 26.69 ± 7.84 years were evaluated. The demographic and ophthalmological characteristics of the groups were summarized in Table 1. There was no statistically significant difference between the groups in terms of age, sex, body mass index, spherical equivalent, axial length, and intraocular pressure (*p* > 0.05).

**Table 1:** Demographic and ophthalmological characteristics of the groups.

	MNSD	Control	*p*-Value
Eye (*n*)	52	52	
Age (years)	26.34 ± 8.14	26.69 ± 7.84	0.686
Male/female	44/8	41/11	0.446
BMI (kg/m^2^)	23.58 ± 4.41	24.32 ± 4.40	0.429
SE	−0.2021 ± 0.62	−0.3798 ± 0.83	0.227
AL (mm)	23.26 ± 0.81	23.50 ± 0.72	0.142
IOP (mm Hg)	16.02 ± 2.73	15.46 ± 2.83	0.318

BMI, body mass index; SE, spherical equivalent; AL, axial length; IOP, intraocular pressure.

The mean subfoveal, nasal, and temporal CT values in MNSD group were 300.67 ± 39.08, 282.06 ± 48.73, 294.71 ± 47.89, respectively, and 292.19 ± 49.22, 282.31 ± 41.70, 282.37 ± 48.27 in the control group, respectively. There was no statistically significant difference in the CT of all three regions between the groups (*p *> 0.05) (Table 2).

**Table 2:** Choroidal thickness measurements at different positions.

	MNSD	Control	*p*-Value
Subfoveal	300.67 ± 39.08	292.19 ± 49.22	0.344
Nasal	282.06 ± 48.73	282.31 ± 41.70	0.762
Temporal	294.71 ± 47.89	282.37 ± 48.27	0.202

The mean foveal thickness of 1000 μm was found to be 249.42 ± 21.83 in the patient group and 257.42 ± 26.25 in the control group, indicating no statistically significant difference in the macular thickness between two groups (*p* > 0.05).

## Discussion

Nasal obstruction due to NSD is one of the most common causes of upper airway obstruction^1^, ^4^ and a risk factor for sleep-disordered breathing.^4^, ^13^ It was mentioned that mechanical upper airway obstructions may lead to chronic hypoxia, hypercarbia, and may cause sympathetic activation and vascular circulatory dysfunction.^14^ Thus, NSD may change the balance of the sympathetic-parasympathetic system and may affect blood flow in choroid and therefore eye health. To study this, we investigated the effects of MNSD on choroidal thickness. Although, CT is not a direct measure of choroidal blood flow, which, is mainly evaluated by laser doppler flowmetry,^15^ in this study we attempted to show the regulation of choroidal blood flow measured by thickness measurements that used EDI-OCT. In the present study, CT was thought to be associated with choroidal blood flow and was compared between the patients with MNSD and healthy controls. However, in this study, no significant differences in terms of choroid thickness measurements in subfoveal, T1 and N1 areas between the groups were observed.

In a meta-analysis of He et al.,^12^ a significant decrease in choroidal thickness of OSAS patients was detected when compared with normal patients. CT of moderate or severe OSAS patients was identified to be thinner than control group, whereas there were similar measurements in the control group versus mild OSAS group. Existence of similar CT measurements between mild OSAS and control group was attempted to be explained by choroidal auto-regulation mechanisms. In mild OSAS patients, the balance between sympathetic and parasympathetic fibers termination in the choroid was thought to be preserved. In our study, it seems that MNSD may have limited effect on choroidal auto-regulation, just like mild OSAS.

In the study of Karaca et al. no correlation between mild, moderate and severe OSAS and choroidal thickness was observed. In this study, all the patients were newly diagnosed; previously diagnosed patients were not included because of mixing effect of the treatment regimen over the results.^11^ They suggested that newly diagnosed patients had healthy parasympathetic innervations of choroid and thus the choroidal blood flow may be preserved in this group of patients. Similarly in our work, in the case of nasal obstruction due to MNSD, the vascular circulation and the balance between sympathetic and parasympathetic regulation may remain stable.

The direct correlation between the severity of nasal obstruction and the severity of sleep-disordered breathing has not yet been clearly established. The results of the studies investigating the effects of nasal obstruction on breathing during sleep are controversial.^16^, ^17^, ^18^, ^19^, ^20^ In the study conducted by Young et al.,^16^ there was an association between nasal congestion and snoring and daytime sleepiness**.** In another study, there was a correlation between airway resistance in the sleep posture of 37 patients and habitual snoring as evaluated by polysomnography and rhinomanometry.^17^ Lofaso et al.^18^ reported that nasal obstruction was an independent risk factor for OSA. In the study conducted by Miljeteig et al.,^19^ 683 patients who applied for sleep polysomnography were divided into three groups according to their nasal resistance, and no significant difference in the apnea and snoring rates was found among the three groups. McNicholas^20^ showed a relationship between the nose and OSA, and suggested that reversible nasal obstruction (allergic rhinitis, iatrogenic) was more associated with sleep-disordered breathing than permanent nasal obstruction such as nasal deviation. In a review investigating all these studies, Georgalas et al.^13^ concluded that nasal obstruction was associated with simple snoring and mild sleep-related breathing disorders, particularly reversible ones. In addition, they proposed that nasal obstruction did not contribute to the vast majority of moderate and severe OSAS patients. Although our study was not intended to reveal the relationship between NSD and sleep-disordered breathing or OSAS, we suggest that our results seem to be relatively consistent with the weaker association of persistent nasal obstruction with sleep-disordered breathing based on the similar effects with mild OSAS on choroidal thickness.

In our study, we also investigated the central macular thickness and no significant difference was found between two groups. The choroid is completely responsible for feeding the foveal part of the eye's macular layer. Therefore, central macular thickness, where the influence of the ischemia hypoxia state is more obvious in the macular center fovea because of the highest oxygen consumption of the photoreceptors in this region.^7^ In the study conducted by Xin et al.,^7^ there was no significant difference in the thickness in the foveal macula between mild and moderate OSAS patients and control group, whereas the severe OSAS group had statistically thinner foveal thickness than the control group.

These results suggest that the nasal obstruction developed in patients with MSND may not lead to significant hypoxia and sympathetic activation, resulting in deterioration of the choroidal blood flow and consequent choroidal thickening.

## Conclusion

This is the first study investigating choroid thickness measurements in patients with MNSD. We found no significant correlation between MNSD and CT. MNSD appears not to cause severe hypoxia and does not lead to significant deterioration in the balance of sympathetic–parasympathetic system, thereby not causing alteration of CT. However, further large-scale studies are required to confirm these findings.

## Conflicts of interest

The authors declare no conflicts of interest.

## References

[1] Ozkececi G., Akci O., Bucak A., Ulu S., Yalim Z., Ayçiçek A. (2016). The effect of septoplasty on pulmonary artery pressure and right ventricular function in nasal septum deviation. Eur Arch Otorhinolaryngol.

[2] Fidan V., Aksakal E. (2011). Impact of septoplasty on pulmonary artery pressure in patients with markedly deviated septum. J Craniofac Surg.

[3] Unlu I., Kesici G.G., Oneç B., Yaman H., Guçlu E. (2016). The effect of nasal obstruction on mean platelet volume in patients with marked nasal septal deviation. Eur Arch Otorhinolarnygol.

[4] Uluyol S., Kılıçaslan S., Gur M.H., Karakaya N.E., Buber I., Ural S.G. (2016). Effects of nasal septum deviation and septoplasty on Cardiac Arrhythmia risk. J Otolaryngol Head Neck Surg.

[5] Nickla D., Wallman J. (2010). The multifunctional choroid. Prog Retin Eye Res.

[6] Margolis R., Spaide R.F. (2009). A pilot stud of enhanced depth imaging optical coherence tomography of the coroid in normal eyes. Am J Opthalmol.

[7] Xin C., Wang J., Zhang W., Wang L., Peng X. (2014). Retinal and choroidal thickness evaluation by SD-OCT in adults with obstructive sleep apnea-hypopnea syndrome (OSAS). Eye (Lond).

[8] Ekinci M., Huseyinoglu N., Cagatay H.H., Keles S., Ceylan E., Gokce G. (2014). Choroidal thickening in patients with sleep apnea syndrome. Neuro-Ophthalmology.

[9] Kara S., Ozcimen M., Bekci T.T., Sakarya Y., Gencer B., Tufan H.A. (2014). Evaluation of choroidal thickness in patients with obstructive sleep apnea/hypopnea syndrome. Arq Bras Oftalmol.

[10] Karalezli A., Eroglu F.C., Kivanc T., Dogan R. (2014). Evaluation of choroidal thickness using spectral-domain optical coherence tomography in patients with severe obstructive sleep apnea syndrome: a comparative study. Int J Ophthalmol.

[11] Karaca E.E., Ekici F., Yalcin N.G., Ciftci T.U., Ozdek S. (2014). Macular choroidal thickness measurements in patients with obstructive sleep apnea syndrome. Sleep Breath.

[12] He M., Han X., Wu H., Huang W. (2016). Choroidal thickness changes in obstructive sleep apnea syndrome: a systematic review and meta-analysis. Sleep Breath.

[13] Georgalas C. (2011). The role of the nose in snoring and obstructive sleep apnea: an update. Eur Arch Otorhinolaryngol.

[14] Olsen K.D., Kern E.B., Westbrook P.R. (1981). Sleep and breathing disturbance secondary to nasal obstruction. J Otolaryngol Head Neck Surg.

[15] Riva C.E., Geiser M., Petrig B.L. (2010). Beijing 100193, PR China Ocular Blood Flow Research Association. Ocular blood flow assessment using continuous laser Doppler flowmetry. Acta Ophtalmol.

[16] Young T., Finn L., Palta M. (2001). Chronic nasal congestion at night is a risk factor for sonoring in a population-based cohort study. Arch Intern Med.

[17] Virkkula P., Bachour A., Hytönen M., Malmberg H., Salmi T., Maasilta P. (2005). Patient- and bed partner-reported symptoms, smoking, and nasal resistance in sleep-disordered breathing. Chest.

[18] Lofaso F., Coste A., d’Ortho M.P. (2000). Nasal obstruction as a risk factor for sleep apnoea syndrome. Eur Respir J.

[19] Miljeteig H., Hoffstein V., Cole O. (1993). The effect of unilateral and bilateral nasal obstruction on sonoring and sleep apnea. Laryngoscope.

[20] McNicholas W.T. (2008). The nose and OSA: variable nasal obstruction may be more important in pathophysiology than fixed obstruction. Eur Respir J.

